# Efficacy of Shaobei injection in the treatment of grade II–III hemorrhoids and the effect on fibulin protein expression: A study protocol of a randomized controlled trial

**DOI:** 10.1097/MD.0000000000027706

**Published:** 2021-11-19

**Authors:** Bin Yue, Yangang Wang, Chunxia Zhang, Yunlong Ding, Zhipeng Liu

**Affiliations:** a Shenyang Coloproctology Hospital, Shenyang City, Liaoning Province, China; b Weifang People's Hospital, Weifang, Shandong Province, China.

**Keywords:** grade II–III, hemorrhoids, randomized controlled trial, Shaobei injection, study protocol

## Abstract

**Background:**

Hemorrhoids are a common and seriously disruptive condition that seriously affects people's lives in terms of treatment. Injection therapy is an effective minimally invasive scheme for the treatment of grade II–III hemorrhoids, but its clinical application is limited by the adverse reactions caused by injection drugs. Some clinical studies have confirmed the efficacy and safety of Shaobei injection as a traditional Chinese medicine extract. However, there is no standard randomized controlled study to verify its efficacy and explore its potential mechanism.

**Methods:**

This is a prospective, randomized, single blind, parallel controlled trial to study the efficacy of Shaobei injection in the treatment of grade II–III hemorrhoids and its effect on the expression of fibulin-3 and fibulin-5 in fibulin protein family. The patients will be randomly divided into a treatment group and control group. The treatment group will be treated with Shaobei injection, and the control group will be treated with rubber band ligation. The observation indexes include: visual analysis scale, postoperative hospital stay, total use of painkillers, fibulin-3 and fibulin-5, hemorrhoids recurrence, and adverse events. Finally, the data will be statistically analyzed by SPASS 18.0 software.

**Discussion:**

This study will compare the efficacy of Shaobei injection with the rubber band ligation method in the treatment of grade II–III haemorrhoids and investigate its effect on the expression of fibulin-3 and fibulin-5 in the fibulin protein family. The results of this study will provide a basis for the clinical use of Paeoniflora injection as an alternative to traditional sclerosing agent in the treatment of grade II–III haemorrhoids.

**Trial registration:** OSF Registration number:DOI 10.17605/OSF.IO/MKVDB

## 1 Introduction

Hemorrhoids are a common clinical disease. At present, the exact prevalence of hemorrhoids is not clear, as most patients have no obvious symptoms in the early stages and do not seek treatment from doctors.^[1]^ A study of patients who underwent routine colorectal cancer screening found that 39% of patients had hemorrhoids, with 55% of these patients reporting no symptoms.^[2]^ Hemorrhoids are more common in people aged 45 to 65 years.^[3]^ Although the exact etiology of hemorrhoids is not clear, hemorrhoids are related to the increase of hemorrhoid venous plexus pressure, such as defecation tension secondary to constipation.^[1]^ Other related factors include obesity, pregnancy, chronic diarrhea, anal sex, cirrhotic ascites, pelvic floor dysfunction, and low fiber diet.^[3,4]^ Although most people have no obvious symptoms in the early stages, as the disease progresses, patients will have to bleed, swelling, prolapse, pain, pruritus and anal discomfort, which seriously affect the quality of life of patients. In addition, repeated bleeding can lead to secondary anemia, hemorrhoids sometimes lead to massive bleeding, requiring emergency hospitalization, and blood transfusion.^[5,6]^

At present, there are many treatment schemes for hemorrhoids, which can be divided into conservative treatment and surgical treatment. Surgical treatment includes rubber band ligation (RBL), injection therapy, hemorrhoidectomy, stapler hemorrhoidectomy etc.^[7]^ For grade II–III hemorrhoids, people prefer minimally invasive surgery, because it means less trauma and shorter recovery time.^[8]^ RBL is recognized as a safe and effective minimally invasive technique. It is one of the most common and simplest minimally invasive therapies for the treatment of symptomatic early hemorrhoids. The literature reports that the recurrence rate of this scheme in the short term is between 12% and 18%, but in the long-term follow-up, up to 42% of patients have intermittent residual symptoms, such as bleeding, pruritus, and anal lump.^[9]^ Therefore, there is still a need to continue to explore a more optimized minimally invasive surgical option.

Injection therapy is another minimally invasive scheme. However, the hardeners used in traditional injection therapy are accompanied by the risks of thrombosis, local tissue ischemia or necrosis, bleeding, and so on. These adverse events limit the clinical application of injection therapy.^[10–12]^ Shaobei injection is a traditional Chinese medicine injection, which is extracted from Wu Mei (Fructus Mume), Wu Bei Zi (*Galla Chinensis*) and Chi Shao (*Radix Paeoniae Rubra*). It can soften and shrink hemorrhoids, inhibit inflammatory reaction and improve pain.^[13]^ In the Chinese hemorrhoid diagnosis and treatment guidelines, Shaobei injection is recommended for the treatment of grade I–III hemorrhoids.^[7,14]^ A retrospective study found that Shaobei injection is effective and safe in the treatment of grade II–III hemorrhoids. Compared with RBL, it has more advantages in reducing postoperative adverse events, hospitalization expenses and the length of time.^[15]^ There is still a lack of prospective clinical studies to validate this conclusion as to whether Shaobei injection is superior to RBL in the treatment of grade II–III haemorrhoids.

In recent years, the mechanism of pathological changes in the anal pad based on elastic fiber lesions has attracted increasing attention, and the relationship between the occurrence and development of hemorrhoids and elastic fibers has attracted more and more attention.^[16]^ The expression of fibulin protein family plays an important role in the formation and metabolism of elastic fibers. Fibulin-3 can form and stabilize basement membrane, elastic fibers, and loose connective tissue.^[17]^ Fibulin-5 not only acts on the synthesis of elastic fibers, but also inhibits neovascularisation.^[18]^ This study will take the classic RBL as the control, through a prospective randomized controlled experiment to explore the efficacy and safety of Shaobei injection in the treatment of grade II–III hemorrhoids, evaluate its effect on the expression of fibulin-3 and fibulin-5 in fibulin protein family, and explore the potential mechanism of Shaobei injection in the treatment of hemorrhoids.

## 2 Materials and methods

### 2.1 Study design

**Figure 1:**
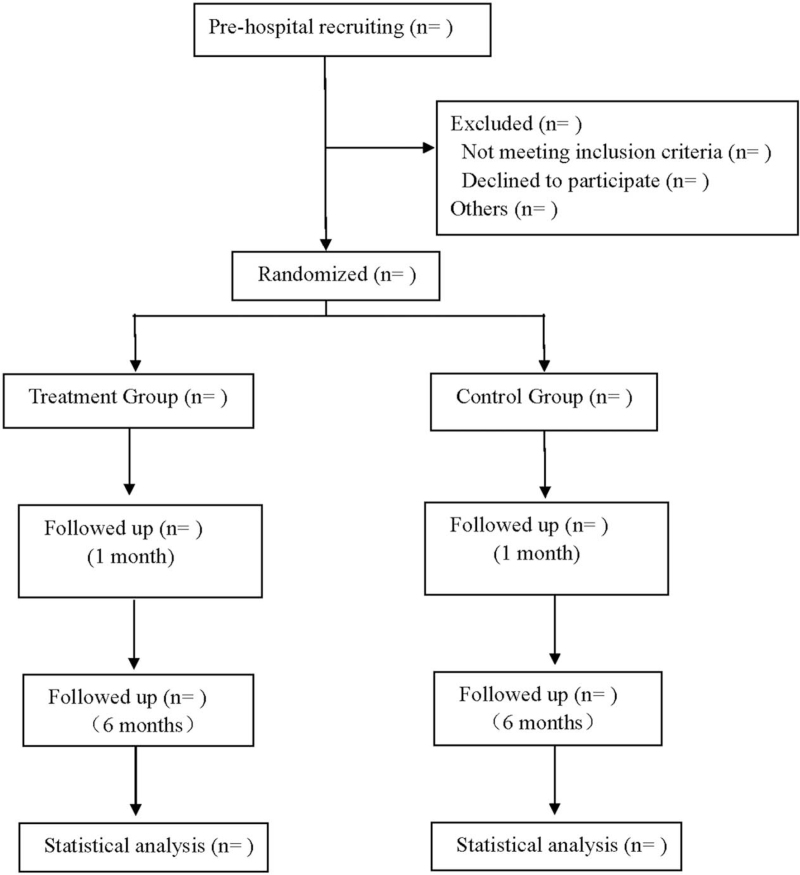
Flow diagram.

This is a prospective, randomized, single blind, parallel controlled trial to study the efficacy of Shaobei injection in the treatment of grade II–III hemorrhoids and its effect on fibulin protein expression. Participants will be randomly divided into a treatment group and control group. The treatment group will receive Shaobei injection and the control group will receive RBL. They will be followed up for 6 months after treatment. Flow diagram is shown in Figure 1. This research scheme follows the latest consolidated standards of reporting trials 2017 and Standard Protocol Items: Recommendations for Interactive Trials 2013 statement.

### 2.2 Ethics and registration

This research plan has been reviewed and approved by our Clinical Research Ethics Committee and will be conducted in strict accordance with the Declaration of Helsinki and Ethical guidelines for Clinical Research, and be supervised by the Ethics Committee. This protocol has been registered in the open science framework (Registration Number: DOI 10.17605/OSF.IO/MKVDB). Patients will be informed in detail of the purpose, plan, and potential risks of the study before the study begins and will sign a written informed consent form after they have agreed to participate in the study.

### 2.3 Patients

#### 2.3.1 Diagnostic basis

For the diagnosis of hemorrhoids, refer to the Guidelines for diagnosis and treatment of hemorrhoids in China (2020),^[7]^ and its classification adopts Goligher classification.^[19]^

#### 2.3.2 Inclusion criteria

(1) Symptomatic grade II or III hemorrhoids diagnosed during preoperative evaluation;
(2) Age ≥18 years and ≤70 years;
(3) The subjects will agree to join the study and sign the informed consent form.

#### 2.3.3 Exclusion criteria

(1) History of hemorrhoid surgery or perianal surgery, with a surgical anastomosis less than 3 cm from the dentate line;
(2) Fecal incontinence or dysdefecation syndrome resistant to drugs;
(3) Combination of other anal diseases (such as anal fissure, anal fistula, etc);
(4) Irritable bowel syndrome with severe constipation or diarrhea;
(5) The presence of any condition that contraindicated surgery;
(6) Pregnant or lactating women.

#### 2.3.4 Abscission criteria and treatment

(1) Those who have a preoperative contraindication to surgery or an intraoperative emergency that, in the judgement of the investigator, warrants discontinuation of the trial and appropriate life-saving treatment;
(2) Those who cannot continue to participate in the study due to subjective or objective factors;
(3) Those who lose many visits and are unable to contact during the follow-up;
(4) Those who are misdiagnosed or accidentally included because they did not meet the inclusion and exclusion criteria.

For the withdrawal or loss of follow-up cases, the researchers should actively take measures to complete the last test as far as possible, so as to analyze its efficacy and safety, and take corresponding treatment measures. All dropped cases should be reported on the case report form (CRF) for the reason for the dropout.

### 2.4 Sample size

The calculation of sample size is based on the mean and standard deviation of visual analogue scale (VAS) scores at rest 24 hours after operation. According to the results of the pilot study, the sample size in the treatment group is 3.71 ± 1.67 and that in the control group is 4.61 ± 1.47. Set up α = 0.025, 1-sided test, β= 0.10. It is calculated by PASS 15.0 software that 66 participants are needed in each group, and the estimated withdrawal rate is 10%. So 74 patients will be included in each group.

### 2.5 Randomization and blinding

We will randomly assign patients who meet the criteria for the treatment group (Shaobei injection group) or the control group (RBL group) in a ratio of 1:1 through a central network-based randomization tool. Random numbers will be generated by independent statisticians who do not participate in the test implementation or statistical analysis with SAS 9.3 software (SAS Institute, Cary, NC). Put the random number into an opaque and sealed envelope, and the patients who meet the standard randomly select the envelope to obtain the random number inside to complete the grouping. Limited by way of intervention, surgeons will know the results of grouping, while patients, anesthesiologists, data analysts, and other research assistants will not know the results of grouping.

### 2.6 Interventions

In order to ensure the consistency of surgical techniques, all patients will be operated by the same surgeon. All patients will use a unified anesthesia scheme. Patients will take the stone position and the surgical site is routinely disinfected. Except for different surgical methods, other treatment schemes are the same.

#### 2.6.1 Treatment group

Shaobei injection (Taifeng Biotechnology Co., Ltd., Henan, China) will be mixed with 1% lidocaine hydrochloride in the volume ratio of 1:1 to prepare injection medicine. Distribution of the hemorrhoid nucleus will be probed under the anoscope to determine the injection site and injected according to the nucleus from small to large. Firstly, 2 mL will be injected under the supra-mucosa of hemorrhoids, and then the needle is injected at the highest point in the center of hemorrhoids. When a feeling of muscular resistance appears, the needle is slowly withdrawn and the prepared injection solution of peony is injected until the haemorrhoid nucleus is full and filled, then gently massage with gauze for 30 seconds, and compressed to stop bleeding. Each haemorrhoid is injected with 3 to 5 mL depending on the size of the haemorrhoid.

#### 2.6.2 Control group

Anal endoscopy will be used to explore the distribution of hemorrhoids, determine the location of ligation hemorrhoids, place the ligation anal endoscope, connect the tail end of the ligation device with the negative pressure aspirator, adjust the negative pressure value of the aspirator to 100 kPa, open the ligation device, inhale the hemorrhoid tissue into the tube, check to ensure that there is no air leakage, tight the elastic line, release the negative pressure suction, and exit the ligation device.

The 2 groups of patients will be wrapped with the same dressing after operation, and the same nursing scheme is adopted after operation. When the pain is unbearable, the patients will be given 0.1 g of nimesulide dispersible tablets orally (Wuhan Changming pharmaceutical, China, GMP H20010730), and the use will be recorded.

### 2.7 Outcomes

(1) The VAS^[20]^ will be used to evaluate patients’ pain changes. The VAS is often used to measure pain intensity.^[21]^ Patients will be asked to mark a point between 0 and 100, where 100 indicate maximum pain (rightmost end) and 0 indicates no pain (leftmost end). VAS scores at rest will be recorded at 4 hours, 12 hours, 24 hours, and 48 hours after operation for both groups. VAS score of patients during defecation will be recorded on the 1st, 2nd, 3^rd^, and 7th day after operation;
(2) The total amount of nimesulide dispersible tablets (tablets) will be taken orally within 7 days after operation;
(3) The patient's postoperative stay in hospital (define as the day of operation started to the key of discharge);
(4) In the first month of postoperative follow-up, the anal pad tissue samples of the patient will be collected according to the wishes of the patient, and fibulin-3 and fibulin-5 in fibulin protein family will be detected by Western blot;
(5) The patients will be followed up in the first month, the third month and the sixth month after operation. The healing or recurrence of hemorrhoids will be evaluated by rectal endoscopy and patient symptoms (including pain, pruritus, bleeding, etc).

### 2.8 Safety evaluation

We will measure blood routine, urine routine, electrocardiogram, liver function, and renal function at baseline and after treatment to evaluate the safety of treatment. Two dedicated researchers will collect any adverse manifestations of patients during the study, including skin pruritus, swelling, necrosis, abnormal bleeding, etc. The details of all adverse events will be recorded in the CRF. The adverse reactions of the 2 groups of patients will be counted at the end of the study. The 2 researchers are unaware of the grouping results.

### 2.9 Data management and quality control

All study data will be collected by 1 to 2 research assistants and recorded in the CRF. The research data will be kept in a separate storage room to protect the confidentiality before, during and after the trial. Access to the database will be limited to the researchers of this research group. The ethics committee of our hospital will regularly monitor the progress of the research and ethical norms, and any change in the scheme will be re-approved by the ethics committee.

### 2.10 Statistical analysis

The data from this study will be statistically analyzed by independent statisticians through SPSS 18.0 software. The Chi square test will be used for counting data; the mean ± standard deviation ($\bar{\text{x}}\pm \text{s}$) will be used for the measurement data, the independent sample *t* test will be used for the normal distribution, and the Mann–Whitney *U* test will be used for the skew distribution. When *P* < .05, the difference will be considered to be statistically significant.

## 3 Discussion

Although there are many treatment schemes for hemorrhoids, the treatment objectives are the same. The guideline points out that the purpose of hemorrhoids treatment is to reduce or eliminate symptoms rather than cure them. Eliminating hemorrhoids symptoms is more meaningful than eliminating hemorrhoids, and should be regarded as the standard of treatment effect.^[22]^ Injection therapy has the advantages of small injury, rapid recovery, and simple operation, which is easy to be favored by doctors and patients. However, there are many kinds of injections, and the safety is not very consistent. We must be very careful in the selection of injections.

Shaobei injection is a traditional Chinese medicine extract, which is mainly composed of citric acid, gallic acid, and paeoniflorin. Citric acid and gallic acid have the effects of acid astringency, astringency and hemostasis, and paeoniflorin has the effects of anti-inflammatory and anti-fibroproliferation.^[23]^ Experimental studies have found that Shaobei injection can effectively reduce local tissue inflammation and edema by inhibiting the release of inflammatory factors by inflammatory cells, and promote the repair and regeneration of mucosal epithelial layer.^[24]^ Clinical studies have found that Shaobei injection therapy can effectively treat grade I–III hemorrhoids, with a cure rate of 100%, the complete atrophy rate of hemorrhoids after 7 days is 95%, and the cure rate after half a year of follow-up is 94.4%.^[25]^ However, there is no standard randomized controlled study to verify these conclusions. This study will take the classic minimally invasive surgery scheme and RBL as the control group to explore the efficacy and safety of Shaobei injection in the treatment of grade II–III hemorrhoids, and we also pay attention to the expression of fibulin-3 and fibulin-5 in fibulin protein family, in order to explore the potential mechanism of Shaobei injection in the treatment of hemorrhoids. Previous studies have found that the recurrence rate of RBL is high.^[9]^ This study will also compare the recurrence of 2 postoperative symptoms through 6-month follow-up, hoping to obtain an optimal treatment scheme.

There are also some deficiencies in this study: firstly, due to the limitations of intervention measures, this study cannot be double-blind, which may cause bias to the results; Secondly, although our follow-up time is 6 months, it may not be enough to pay attention to the recurrence indicators of patients; Thirdly, we need to detect the expression of fibulin-5 protein by taking the tissue expression of anal pad again, which may lead to the loss of follow-up of some patients.

## Author contributions

**Conceptualization:** Bin Yue and Yangang Wang.

**Data curation:** Chunxia Zhang and Yunlong Ding.

**Formal analysis:** Bin Yue and Yunlong Ding.

**Funding acquisition:** Zhipeng Liu.

**Software:** Yangang Wang and Chunxia Zhang.

**Supervision:** Chunxia Zhang and Yunlong Ding.

**Writing – original draft:** Bin Yue and Yangang Wang.

**Writing – review & editing:** Bin Yue and Zhipeng Liu.
